# Aerated Waters

**Published:** 1905-02-11

**Authors:** 


### AERATED WATERS.

THE Secretary of Aerators, Limited, writes:—Sir,—We have read with much interest the article on "Aerated Waters" in your issue of January 28th, 1905, but think the sentence in which you speak of the "contamination" of the pure element by carbonic acid gas is apt to be misleading. We presume that your intention was to condemn the cheap aerated drinks made from dirty water and impure carbonic acid gas, which are largely sold amongst the poorer classes. The beneficial effects of the impregnation of good wholesome water with pure $CO_2$ have frequently been recognised in your columns.

